# Congenital Zika syndrome and neuroimaging findings: what do we know so far?

**DOI:** 10.1590/0100-3984.2017.0098

**Published:** 2017

**Authors:** Bruno Niemeyer de Freitas Ribeiro, Bernardo Carvalho Muniz, Emerson Leandro Gasparetto, Nina Ventura, Edson Marchiori

**Affiliations:** 1 Masters Student, MD, Neuroradiologist at the Instituto Estadual do Cérebro Paulo Niemeyer, Rio de Janeiro, RJ, Brazil.; 2 Full Member of the Colégio Brasileiro de Radiologia e Diagnóstico por Imagem (CBR), MD, Neuroradiologist at the Instituto Estadual do Cérebro Paulo Niemeyer, Rio de Janeiro, RJ, Brazil.; 3 PhD, MD, Neuroradiologist, Head of the Instituto Estadual do Cérebro Paulo Niemeyer, Rio de Janeiro, RJ, Brazil.; 4 PhD, MD, Neuroradiologist at the Instituto Estadual do Cérebro Paulo Niemeyer, Rio de Janeiro, RJ, Brazil.; 5 Full Professor at the Universidade Federal do Rio de Janeiro (UFRJ), Rio de Janeiro, RJ, Brazil.

**Keywords:** Magnetic resonance imaging, Computed tomography, Zika virus, Congenital Zika syndrome, Congenital infection, Ressonância magnética, Tomografia computadorizada, Vírus Zika, Síndrome congênita pelo vírus Zika, Infecção congênita

## Abstract

Although infection with the Zika virus was first recognized in 1942, it received little attention until 2007, when a true pandemic spread throughout Africa, Asia, and the Americas. Since then, numerous forms of central nervous system involvement have been described, mainly malformations related to congenital infection. Although the neuroimaging findings in congenital Zika syndrome are not pathognomonic, many are quite suggestive of the diagnosis, and radiologists should be prepared to interpret such findings accordingly. The objective of this article is to review the computed tomography and magnetic resonance imaging findings in congenital Zika syndrome.

## Abstract (pt)

A infecção pelo vírus Zika, apesar de conhecida desde 1942, apresentou destaque somente a partir de 2007, quando uma verdadeira pandemia se espalhou pela África, Ásia e Américas. Durante este período, numerosas formas de acometimento do sistema nervoso central têm sido descritas, principalmente as malformações relacionadas a infecção congênita. Apesar de os achados de neuroimagem na síndrome congênita pelo vírus Zika não serem patognomônicos, muitos são bastante sugestivos, devendo o radiologista estar preparado para saber interpretar e sugerir o diagnóstico. O objetivo deste artigo é revisar os achados de tomografia computadorizada e ressonância magnética da síndrome congênita pelo vírus Zika.

## INTRODUCTION

Flaviviruses are among the most important emerging viruses known to man, being transmitted by mosquitoes and ticks. The Zika virus (ZIKV) pandemic is the most recent of the arthropod-borne viral diseases, following on the heels of dengue, West Nile virus, and chikungunya, for which outbreaks were recorded in 1990, 1999, and 2013, respectively^(1)^. ZIKV is an arbovirus of the family Flaviviridae, composed of ribonucleic acid, which was discovered in Uganda in 1947, although the first case in humans was not reported until 1952^(1-8)^. Typically, it occurs in tropical and subtropical areas of the world, mainly in Africa and Asia, the two major lineages (Asian and African) originating from a common ancestor^(1-8)^. Like other arboviruses, ZIKV presents many barriers to the accumulation of mutations, as a consequence of double replication in mammalian and invertebrate hosts, and the rate of fixation of its mutations is therefore relatively slow^(5)^.

Beginning in 2007, ZIKV, which had until then been confined to a narrow equatorial zone in Africa and Asia, began to become more widespread, affecting Micronesia; in the 2013–2014 period, there was a ZIKV epidemic in French Polynesia and New Caledonia. Since then, there has been a progressive expansion of the virus, cases of ZIKV infection having been reported in South America, Central America, and the Caribbean Islands^(1,4)^, the displacement of people and the presence of vectors being important factors for its dissemination.

The first autochthonous ZIKV transmission in Brazil occurred in May 2015^(4,8)^, with probable dissemination from the Pacific region, given that phylogenetic studies have shown high similarity (99.7% for nucleotides and 99.9% for amino acids) with the virus circulating among islands in the Pacific Ocean^(2)^. Brazil was the Latin American country most affected by ZIKV, approximately 1,500,000 cases being reported in 2015 and 2016^(3)^. Global warming and climate change, together with the El Niño phenomenon, might have contributed to accelerating the spread of the virus and its vector, additional factors including the low socioeconomic status and lack of awareness of the population, as evidenced by the fact that the epidemic was more intense in impoverished areas of the northern and northeastern regions of the country, especially in the states of Pernambuco, Bahia, and Paraíba^(3)^.

During epidemics, various forms of central nervous system (CNS) involvement associated with ZIKV infection have been reported, such as meningoencephalitis, Guillain-Barré syndrome, and acute disseminated encephalomyelitis^(2,3,5,7,9)^. Simultaneously, numerous cases of CNS malformations potentially related to congenital ZIKV infection, characterizing congenital Zika syndrome, have gained prominence in the scientific community, having been widely documented by computed tomography (CT) and magnetic resonance imaging (MRI).

Although the neuroimaging findings in congenital Zika syndrome are not pathognomonic, many are quite suggestive of the diagnosis, and radiologists should be prepared to recognize those findings, interpret them, and propose the diagnosis. The objective of this article is to review the CT and MRI findings in congenital Zika syndrome. To that end, we searched the PubMed database using the following search terms: “Zika virus”; “congenital Zika virus infection”; “zika neuroimaging”; “zika magnetic resonance imaging”; “zika computed tomography”; and “zika ultrasound”. We analyzed articles indexed up through May 2017.

## ZIKV TRANSMISSION

The transmission of ZIKV occurs mainly by the bite of mosquitoes of the genus *Aedes*, which are common in the tropics and are also recognized vectors of dengue fever, yellow fever, and chikungunya. Although the main vector of ZIKV transmission is *A. aegypti*, other *Aedes* species, such as *A. albopictus*, *A. africanus*, *A. luteocephalus*, *A. vittatus*, *A. furcifer*, *A. hensilli* and *A. apicoargenteus*, can transmit the virus, as can mosquitoes of the genera *Anopheles*, *Eretmapodites*, *Culex*, and *Mansonia*^(1,3,5,8)^.

*Aedes aegypti* is widely distributed in the Americas, usually lives in close proximity to people and their homes, lays its eggs in still waters, as well as in water that collects in buckets, flowerpots, empty pipes, and other containers, and bites mainly during the daytime^(3,4,7,8)^.

The infection cycle begins when *Aedes* species ingest ZIKV-infected blood, which initiates a process of viral replication in the epithelial cells of the midgut and migration of the virus to the salivary glands of the mosquito; after 5–10 days, the mosquito is able to transmit the virus to healthy individuals^(3,4)^. Other forms of transmission, such as blood transfusion, sexual (oral, anal, or vaginal) transmission, vertical (transplacental or perinatal) transmission, and transmission via urine, have been described^(1,2,4,8)^. Because most patients infected with ZIKV are asymptomatic, there is an extremely high risk of blood donors acting as a source of transmission in endemic areas^(8)^. Other suspected routes of ZIKA transmission include monkey bite, organ transplantation, and hemodialysis^(3)^. In kidney transplantation, the risk of ZIKV infection should be considered if the donor is a resident of or is returning from an endemic area, given ZIKV can be detected in the urine of people infected for more than 30 days^(3)^. Despite the fact that the viral particle has been isolated in breast milk, there is as yet no evidence of transmission through breastfeeding, although that route of transmission has been described for other flaviviruses^(3,5,10)^.

## CLINICAL ASPECTS OF ZIKV INFECTION

In 75–80% of cases, ZIKV infection is asymptomatic^(3)^. In symptomatic cases, after an incubation period that typically lasts for 3–12 days, the most common manifestation is a self-limiting profile—low-grade fever (37.8–38.5ºC); headache; muscle aches; pain in the small joints of the hands and feet; non-purulent conjunctivitis; ocular pain; prostration; and pruritic maculopapular rash—similar to that of dengue, albeit milder and typically evolving to regression of the symptoms in 2–7 days^(1,3,4)^. Hematological and biochemical parameters are usually normal^(1,3)^. After the first infection, the person develops immunity and will not develop the disease if again exposed to the virus^(2-5)^.

Although severe and fatal forms of ZIKV are quite rare, cases of Guillain-Barré syndrome, meningoencephalitis, and acute disseminated encephalomyelitis have been reported^(1,2,8,9,11)^, such cases having been attributed to the high degree of tropism of ZIKV for the CNS^(8)^.

## DIAGNOSIS

Fauci et al.^(12)^ reported that the diagnosis of ZIKV infection can be made mainly on the basis of the clinical findings. However, the authors stated that this is only applicable in areas in which there is a current ZIKV epidemic and other diseases with a similar clinical presentation, such as dengue and chikungunya, are not endemic.

The diagnosis of ZIKV can be confirmed through amplification of the viral genome by reverse transcriptase-polymerase chain reaction (RT-PCR) in samples of blood, saliva, urine, cerebrospinal fluid and amniotic fluid, although the RT-PCR procedure is expensive and subject to contamination. In addition, due to the kinetics of ZIKV viremia, the timing of the investigation depends on the material collected^(1,3,8)^: in blood and saliva, RT-PCR can be performed only up until the seventh day after the onset of symptoms; in urine, the virus can be detected for more than 30 days after the onset of symptoms; and, in male patients, the virus can be detected in semen within the first 3–8 weeks after the onset of symptoms. Serological tests for the detection of immunoglobulin M antibodies against ZIKV are usually conducted 4–5 days after the onset of symptoms, and those antibodies can remain detectable for an additional 2–3 months, as has been reported for other flaviviruses^(2-4,8)^.

## TREATMENT

Because ZIKV infection is usually self-limiting, the treatment consists of increased fluid intake and rest. Pharmacological treatment should be restricted to symptom relief^(3)^.

There are as yet no vaccines or antiviral therapies for ZIKV infection. Therefore, preventive measures, focusing on vector eradication—such as reducing the number of water reservoirs available for oviposition and using insecticides—and on mosquito bite avoidance—such as the use of repellents—are fundamental as the best form of controlling the disease^(3,8)^. It is also recommended that to that travelers returning from endemic areas to nonendemic areas be advised to use repellents for a minimum of 14 days, in order to prevent local mosquitoes from getting the virus^(3)^. Patients with clinical signs of the disease and individuals returning from endemic areas should also be advised to use condoms or abstain from sex, especially if their partner is pregnant^(2,3)^. Some health authorities also recommend that women avoid becoming pregnant during ZIKV epidemics and that those who are pregnant or intending to become pregnant avoid traveling to endemic areas^(3)^. Pregnant women who are symptomatic should be tested for ZIKV by RT-PCR, as should those in whom there is evidence of microcephaly on ultrasound. In the latter cases, follow-up ultrasound examinations should be performed every 3–4 weeks^(2)^.

Recently, the vector control group of the World Health Organization discussed the use of genetically modified mosquitoes for the control of *A. aegypti*. By competing with wild *A. aegypti* males, the male OX513A mosquito was successful in controlling dengue in Brazil^(2)^.

## CAUSALITY OF MALFORMATIONS AND CONGENITAL ZIKV INFECTION

The cautious approach to implicating ZIKV as a cause of birth defects is not surprising, given that the last epidemic of birth defects caused by an infectious pathogen (the rubella virus) occurred more than 50 years ago^(13)^. In addition, no flaviviruses have been definitively demonstrated to be a cause of birth defects in humans and there have been no reports of adverse pregnancy events during previous ZIKV outbreaks^(13,14)^.

Two methods have been used in order to identify potential teratogens: the identification of the combination of a rare exposure and presentation of a rare defect, as was the case for rubella virus, which was identified by an ophthalmologist after noticing a characteristic form of cataract in children born to mothers with rubella infection during pregnancy^(13,15)^; and the identification of a causal relationship, which can be achieved through the use of epidemiological studies, as in the case of valproic acid, which was identified as a teratogen after a case-control study demonstrated a 20-fold increase in the incidence of spina bifida when valproic acid was administered during the first trimester of pregnancy^(13,16)^. In 1994, Thomas Shepard, a pioneer in the field of teratology, proposed seven criteria, including those previously cited, to confirm teratogenicity^(17)^. According to the Shepard system, causality is established through a rare exposure-rare defect approach—when criteria 1, 3, and 4 (as defined below) are met—or through an epidemiological approach—when criteria 1, 2, and 3 (as defined below) are met^(13)^.

In the Shepard system, criterion 1 is exposure to a given agent at a critical time during the course of the pregnancy. Microcephaly and other brain abnormalities that have been observed in many infants are consistent with infection occurring in the first trimester or at the beginning of the second trimester of pregnancy. An analysis of the time to biochemical confirmation of ZIKV transmission in certain Brazilian states and of the increased incidence of microcephaly identified the first trimester of pregnancy as the critical period of ZIKV infection^(18)^.

Criterion 2 in the Shepard system is the existence of at least two high-quality epidemiological studies supporting the association. Data from Brazil and French Polynesia demonstrated temporal and geographic associations between ZIKV infection and the subsequent appearance of infants with congenital microcephaly^(18-30)^. In one of those studies, conducted during the outbreak in Brazil, 88 pregnant women with a skin rash were tested for ZIKV RNA. Of the 72 who tested positive, 42 underwent ultrasound. Among those 42 women, fetal abnormalities were observed in 12 (29%). The 16 women who tested negative did not present abnormalities on the ultrasound examination^(21)^. Although those studies provided important evidence of a causal relationship, they presented limitations, as cited by their authors^(18,19,21)^, including a lack of control for confounding factors and a relatively small number of cases. Therefore, criterion 2 is not strictly met.

Shepard system criterion 3 is a specific defect or syndrome occurring after exposure to a given teratogen. In fact, many fetuses and infants with congenital ZIKV infection have presented a characteristic appearance, including severe microcephaly, intracranial calcifications and other brain abnormalities, sometimes accompanied by ocular alterations, cutis verticis gyrata (excessive scalp skin), arthrogryposis, and talipes equinovarus (clubfoot), leading some authors to coin the term “congenital Zika syndrome” to describe the presentation^(22,23)^. In addition, cutis verticis gyrata is not a typical finding in other forms of microcephaly.

Criterion 4 in the Shepard system is an association between a rare exposure and the subsequent appearance of a rare defect, the rationale of which is that it is unlikely that two rare events will occur in conjunction, especially with the appropriate chronology to suggest causation. Microcephaly is a rare defect estimated to occur in 6 out of 10,000 live births in the United States^(13)^. Although exposure to ZIKV was not uncommon among women residing in Brazil during the outbreak, there were reports of adverse events in fetuses among female travelers who were in endemic areas for only a short time, constituting a rare exposure^(24,25)^. In one report, a European woman presented fever and a skin rash at the end of week 13 of gestation, while in the state of Rio Grande do Norte, in northeastern Brazil. Prior to that, the woman had undergone a number of ultrasound examinations, none of which had shown any abnormalities. However, ultrasound performed at 29 weeks of gestation revealed multiple cerebral malformations. The fetus was aborted, and an autopsy was conducted. All organs were examined, as were the placenta and umbilical cord. The findings included microcephaly, pachygyria/lissencephaly, and hydrocephalus, together with multifocal dystrophic calcifications in the cortex and subcortical white matter, as well as signs of local inflammation and placental calcifications. There were no relevant pathological abnormalities in the remaining organs, and RT-PCR of the brain tissue was positive only for ZIKV, no other flaviviruses being identified. The ZIKV strain identified bore a high (99.7%) resemblance to the Asian lineage identified in the outbreak that occurred in French Polynesia in 2013^(25)^.

Shepard system criterion 5 is the finding of teratogenicity in animal models. Animal studies of ZIKV have demonstrated not only its neurotropism but also its teratogenic potential^(26-29)^.

Criterion 6 in the Shepard system is that the association makes biological sense. Such sense has clearly been demonstrated, given that other viral agents have similar effects, as well as the fact that studies have shown that ZIKV has a high degree of tropism for and a deleterious effect on fetal brain tissue^(24,25)^.

The criterion 7 in the Shepard system is used only for exposure to medications and chemicals. Therefore, it does not apply to exposure to infectious agents.

In view of the above, according to the Shepard system, there is strong evidence that ZIKV is a teratogen.

## MANIFESTATIONS OF CONGENITAL ZIKV INFECTION

The effects of intrauterine ZIKV infection are more severe when they occur in the first or second trimesters of pregnancy, especially in the first trimester, ranging from fetal death to various congenital abnormalities, which include cutis verticis gyrata (of the scalp, neck, or both), low birth weight, polyhydramnios, anasarca, arthrogryposis, and hearing loss, as well as ocular and CNS malformations with craniofacial manifestations^(3,25,30-33)^.

Of all the congenital manifestations of ZIKV infection, the one that has had the greatest impact is microcephaly, which has been well documented, in Brazil and French Polynesia, and has been associated with the Asian lineage of the virus. During the ZIKV epidemic in Brazil (from March 2015 to February 2016), there was a 20-fold increase in the incidence of microcephaly in comparison with previous years^(3)^.

In the literature, there is no consensus regarding the definition of microcephaly. Microcephaly is a brain growth disorder in which the head circumference is smaller than that expected for age, gender, and race; the cut-off value can be the 3rd or 5th percentile or 2–3 standard deviations below the mean^(31-33)^. It can be caused by numerous conditions, including genetic disorders and infectious diseases (rubella, cytomegalovirus infection, toxoplasmosis, herpes virus infection, HIV infection), as well as exposure to alcohol, drugs, or other toxic substances present in the environment. Clinically, patients with microcephaly present severe neurological impairment, common symptoms being hypertonia, spasticity, and seizures^(13,15-17,33,34)^.

The pathogenesis of ZIKV microcephaly is not fully understood, although it is believed to begin with placental infection. In the placental tissue of pregnant women infected with ZIKV, Noronha et al.^(29)^ detected viral proteins in Hofbauer cells and some histiocytes in the intervillous spaces, suggesting that ZIKV damages the placental barrier, inducing chronic placentitis^(29)^, as has also been documented in rats^(28)^. As gestation progresses, the virus disseminates to the fetal brain, where it preferentially infects neuronal progenitor cells, decreasing their viability and impeding their growth, thus inhibiting cell proliferation, cell differentiation, and neuronal apoptosis, with consequent thinning of the cortex and macroscopic signs of microcephaly^(3,30)^. It has also been suggested that the placental inflammatory process can act in synergy with cerebral ZIKV infection in the genesis of brain malformations^(32)^.

Despite the neurotropism of ZIKV, there have been no reports of microcephaly in infected children soon after birth. That is probably attributable to the low infective potential of ZIKV in developed neural cells^(3,32,33)^.

## NEUROIMAGING FINDINGS IN CONGENITAL ZIKA SYNDROME

In CT and MRI studies of congenital Zika syndrome, the findings most commonly reported are a reduction in head circumference and a dramatic reduction in brain volume, both of which are indicative of microcephaly and are more common when the infection occurs in the first trimester of pregnancy, with a risk of 1–13%^(34-41)^. Microcephaly can be asymmetric. The condition is mild in 25% of cases and moderate-to-severe in 75%^(35)^.

**Figure 1:**
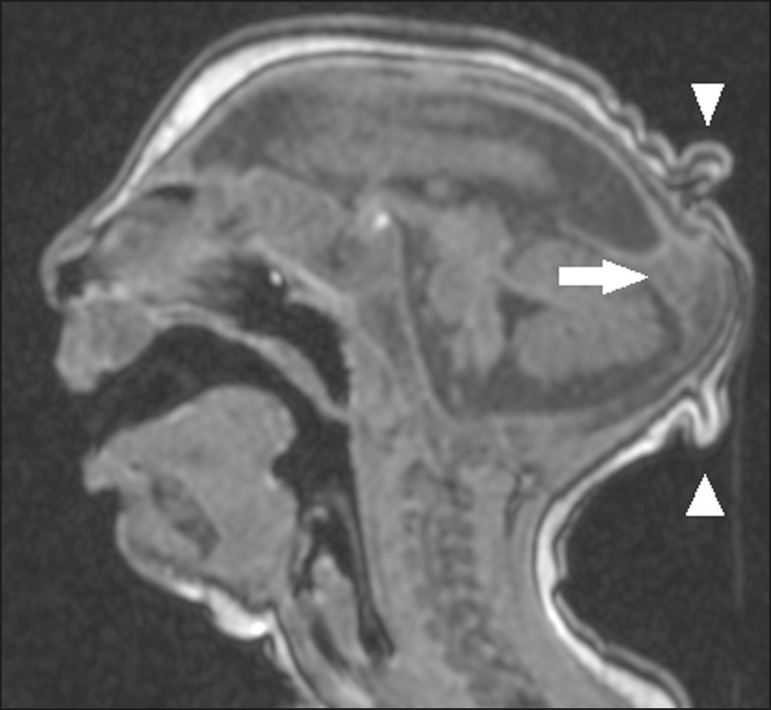
A 3-month-old patient. Sagittal T1-weighted MRI slice, without contrast, showing craniofacial disproportion with a microcephalic aspect, together with occipital prominence and cutis verticis gyrata (arrowheads). Note also the confluence of the enlarged dural venous sinuses and the heterogeneous material (arrow).

An uncommon appearance of the skull, a finding that is common in congenital Zika syndrome and uncommon in microcephaly of other causes, is characterized by a collapsed aspect of the skull cap, with overlapping sutures and prominent occipital bones, creating a “shelf” appearance, commonly accompanied by excessive, folded skin on the scalp (Figure 1). That presentation could be due, in part, to the continued growth of the skull and skin while the size of the brain regresses, or even, at some point, collapse of the skull, which previously had larger dimensions due to cerebral ventriculomegaly^(34-40)^. One unusual finding that occurs in some newborns and suggests the latter hypothesis is herniation of the orbital fat to the skull, which could be secondary to the abrupt deformation of the skull, rather than direct infection of the eye, as occurs in other congenital infections^(34)^.

**Figure 2:**
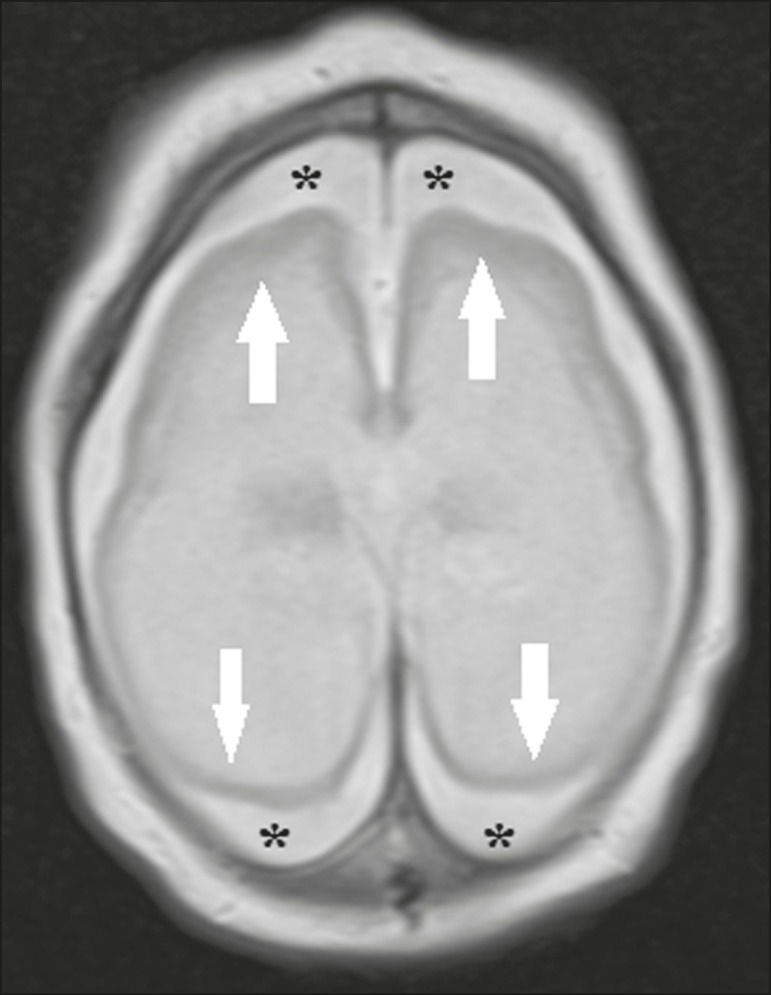
A 2-month-old patient. Axial T2-weighted MRI slice showing marked simplification of the gyral pattern, with agyria (arrows). Note also the diffuse increase in the extra-axial CSF space (asterisks).

Abnormalities of cortical development are frequent findings, occurring in 94–100% of cases, commonly presenting as agyria-pachygyria (Figure 2), and probably varying according to the stage of cortical development in which the infection occurs^(34-43)^. The involvement is usually diffuse, predominantly occurring in the frontal, insular, and parietal lobes, with varying degrees of severity; it is frequently accompanied by wide sylvian and interhemispheric fissures, as well as by an increase in the extra-axial cerebrospinal fluid (CSF) space, the last being associated with a loss of brain volume and impaired CSF reabsorption^(34-43)^. Gray matter heterotopia is rare^(38)^.

**Figure 3:**
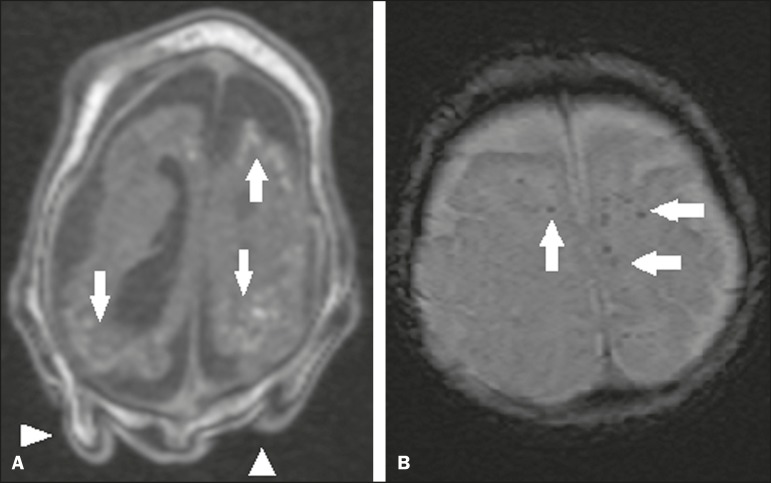
A: A 2-month-old patient. Axial T1-weighted MRI slice, without contrast, showing multiple punctate foci of hyperintensity at the cortical-subcortical junction of the frontal and parietal lobes, indicative of calcifications (arrows). Note also the cutis verticis gyrata (arrowheads). B: A 6-month-old patient. Axial susceptibility-weighted MRI sequence showing foci of hypointensity at the cortical-subcortical junction, affecting both hemispheres of the brain and more conspicuous in the frontal regions, because of the presence of calcifications (arrows).

**Figure 4:**
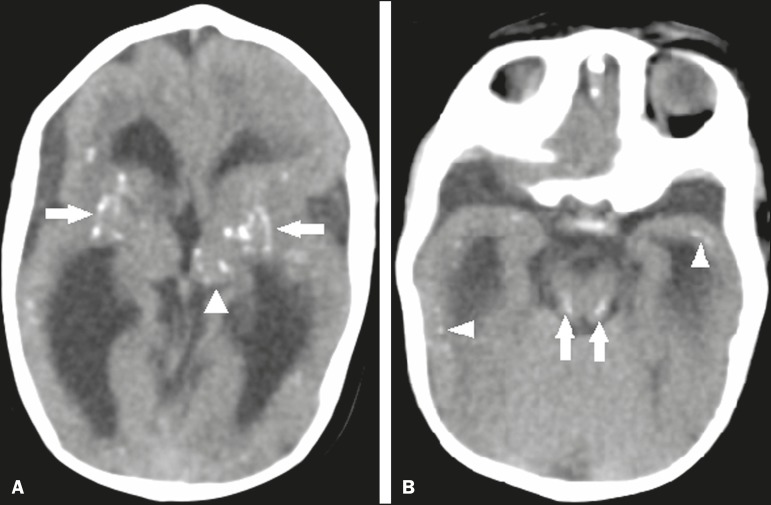
A: A 5-month-old patient. Axial CT scan, without contrast, showing foci of calcification in the basal nuclei (arrows) and left thalamus (arrowhead). B: A 5-month-old patient. Axial CT scan, without contrast, showing calcifications in the dorsolateral regions of the mesencephalic tegmentum (arrows), as well as at the cortical-subcortical junction in the temporal lobes (arrowheads).

Calcifications are common in congenital Zika syndrome, occurring in 88–100% of patients; unlike classic infections (toxoplasmosis, rubella, cytomegalovirus, herpes simplex, HIV, and syphilis), in which calcifications are periventricular and cortical, ZIKV infection tends to involve the cortical-subcortical junction (Figure 3); one possible explanation is that ZIKV infection has a vascular component, given that other processes that mainly affect this region are associated with vascular alterations^(34-43)^. As illustrated in Figure 4, there are other sites that present calcifications in ZIKV infection^(34-40)^, which are, in descending order of frequency, the following: the basal nuclei and thalamus (in 29–65% of cases); the periventricular region (in 14–65%); the cortical region (in 14–24%); and the infratentorial region (in 4–18%). It is noteworthy that periventricular and cortical involvement are more common in newborns in whom there is a significant loss of brain parenchymal volume; therefore, the precise location of calcifications in such newborns is difficult to determine^(34)^. According to Oliveira-Szejnfeld et al., infratentorial calcifications are present in more severe manifestations, being accompanied by brainstem malformation, aqueduct stenosis, and secondary hydrocephalus^(34)^.

There are inherent differences between CT and MRI, the former presenting greater sensitivity in the detection of calcifications even when compared with susceptibility-weighted MRI sequences, whereas MRI presents a greater capacity for characterizing cortical abnormalities and the development of the corpus callosum^(34-40)^.

**Figure 5:**
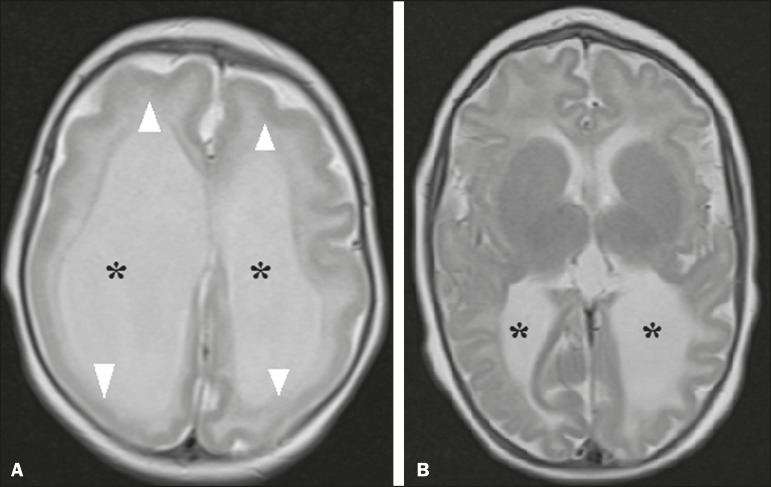
A: A 7-month-old patient. Magnetic resonance imaging, T2, axial section showing diffuse enlargement of the lateral ventricles (asterisks) and simplification of the gyral pattern (arrowheads). B: An 8-month-old patient. Axial T2-weighted MRI slice showing asymmetric dilation of the posterior portions of the lateral ventricles (asterisks), constituting a colpocephaly configuration.

Enlargement of the lateral ventricles, as depicted in Figure 5, is common, occurring in 94–100% of cases; in most cases, it is moderate-to-pronounced and symmetric; it is accompanied by septations in 10–29% of cases, and occipital horns are commonly seen, sometimes making it difficult to distinguish periventricular cysts^(34-43)^. It should also be borne in mind that the presence of ventriculomegaly is directly related to a reduction in brain volume and can cause the head circumference to remain normal or even increase^(34-40,43)^.

**Figure 6:**
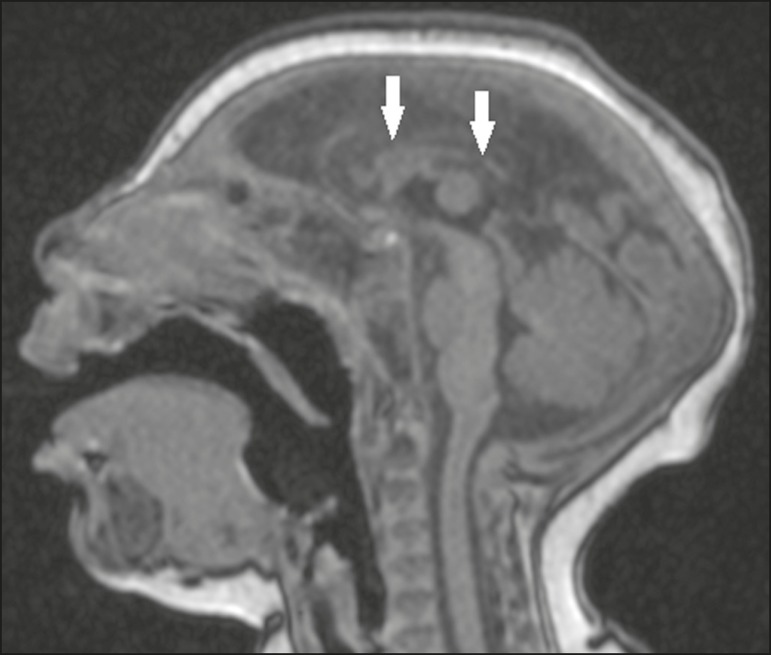
A 3-month-old patient. Sagittal T1-weighted MRI slice, without contrast, showing diffuse tapering of the corpus callosum (arrows). Note also the craniofacial disproportion with a microcephalic aspect.

Hypoplasia, dysgenesis, and agenesis of the corpus callosum are often seen in congenital Zika syndrome (Figure 6), reportedly occurring in 75–94% of cases and presenting a direct correlation with parenchymal damage^(34-43)^. Other associated abnormalities are hippocampal malformation and thickening of the fornix^(34-40)^.

**Figure 7:**
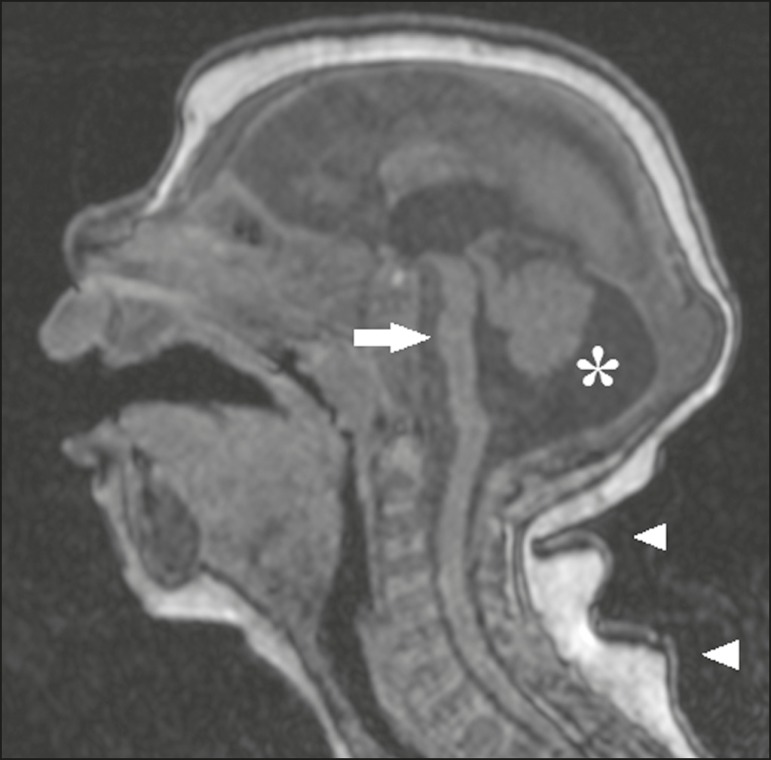
A 2-month-old patient. Sagittal T1-weighted MRI slice, without contrast, showing hypoplasia of the pons (arrow), with loss of the usual convexity. Also shown are an enlarged cisterna magna (asterisk), reduced cerebellar volume, and excessive skin in the nuchal region (arrowheads).

**Figure 8:**
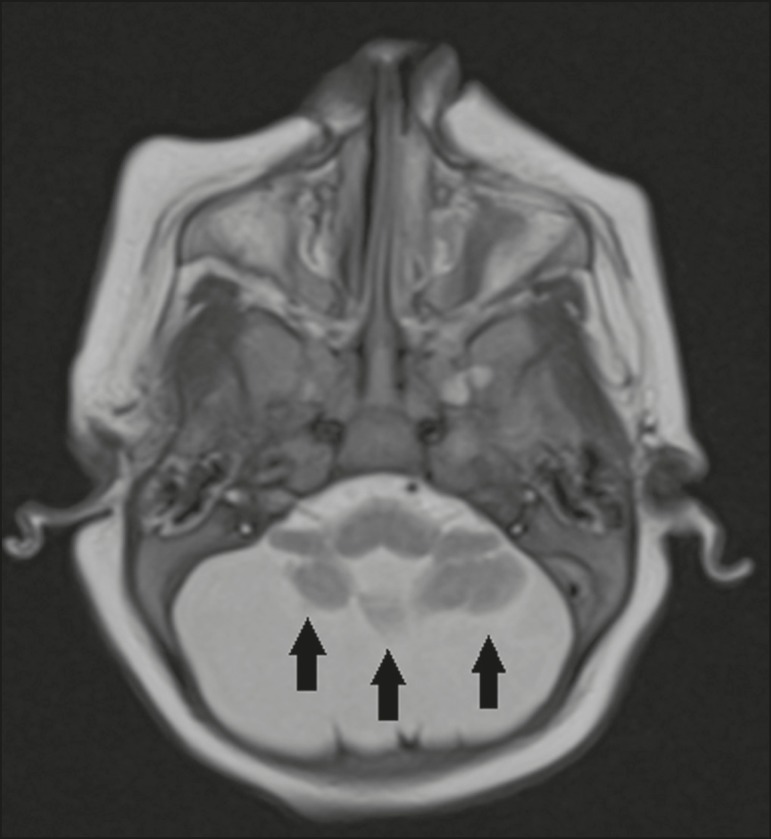
A 2-month-old patient. Axial T2-weighted MRI slice showing diffuse, symmetric cerebellar hypoplasia (arrows), with prominence of the CSF spaces in the posterior fossa.

Brainstem abnormalities, which have been reported in 21–70% of patients with congenital ZIKV infection, are characterized by a tapered, atrophic encephalic trunk with preferential involvement of the pons (Figure 7), are commonly associated with more severe conditions and can be related to synergism between the reduction in the number of descending fibers and the direct effects of the virus^(34-43)^. Other abnormalities of the posterior fossa include cerebellar hypoplasia, which is usually diffuse and symmetric (Figure 8), occurring in 27–82% of cases, and an enlarged cisterna magna, the frequency of which increases in proportion to the severity of the infection, although there is no direct correlation between enlarged cisterna magna and the occurrence of cerebellar hypoplasia^(34-43)^.

Alteration of the white matter MRI signal secondary to a delay in myelination is seen in 88–100% of cases of congenital ZIKV infection. Confluence of the enlarged dural venous sinuses with heterogeneous material (Figure 1), which can correspond to a thrombus or a hematocrit effect due to dehydration with hemoconcentration, is seen in 28–53% of cases^(34,35,38,41,42)^.

Currently, there are three severity spectra in congenital Zika syndrome^(41)^: with microcephaly at birth, presenting all of the abnormalities described in the literature and with a symmetric appearance; with postnatal microcephaly, the presentation of which is comparable to that of microcephaly at birth, minus the calcifications outside the cortical-subcortical junction and the agyria; and without microcephaly, which presents with calcifications restricted to the cortical-subcortical junction, areas of pachygyria, a delay in myelination, and slight ventricular enlargement, with an asymmetric appearance. In addition, as proposed by Aragão et al.^(41)^, the presence of polymicrogyria mainly in the frontal lobes is seen only in patients without microcephaly or with postnatal microcephaly.

**Figure 9:**
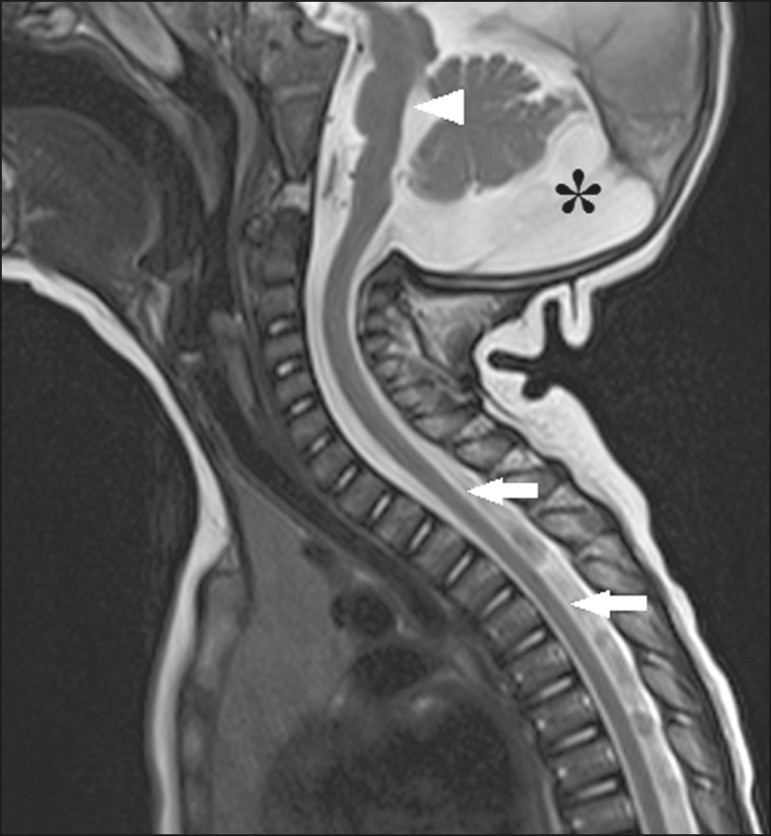
A 14-month-old patient. Sagittal T1-weighted MRI slice showing tapering of the dorsal medulla (arrows) in a congenital Zika syndrome patient without arthrogryposis. Also shown are pontine hypoplasia (arrowhead) and an enlarged cisterna magna (asterisk).

Recently, spinal and nerve root changes have been described in congenital Zika syndrome, the severity of those changes presenting an apparent correlation with arthrogryposis, defined as contracture of two or more joints from birth^(42)^. Visual inspection of T2-weighted MRI sequences in the sagittal and axial planes has revealed a significant reduction in the thickness of the entire spinal cord, accompanied by a significant reduction in the thickness of the anterior roots of the medullary cone, in patients with congenital Zika syndrome and arthrogryposis. However, patients who do not present such joint changes have been found to show a reduction in spinal thickness only in the dorsal region and only discrete tapering of the anterior roots of the medullary cone (Figure 9)^(42)^. Clear involvement of the descending anterior medullary tracts, with apparent preservation of ascending posterior tracts, has also been seen, as reported by Mlakar et al.^(25)^.

Aragão et al.^(42)^ showed that arthrogryposis correlates with greater severity of brain damage, with a greater number of cerebral calcifications and a greater chance of infratentorial calcifications, as well as a greater chance of hypoplasia of the brainstem and cerebellum. In addition, all of the cases in which there was arthrogryposis have presented with pachygyria and an absence of polymicrogyria, which could indicate that congenital Zika syndrome with arthrogryposis occurs in the earlier stages of fetal development, because pachygyria results from failure of neuronal migration and of cortical organization between weeks 12 and 16 of gestation, whereas polymicrogyria occurs around week 20^(42)^.

## CONCLUSION

Fetal infection with ZIKV causes severe CNS developmental abnormalities. Although the neuroimaging findings are not pathognomonic, they can be suggestive of congenital Zika syndrome when the clinical and biochemical data are consistent with the diagnosis.

The main findings in congenital Zika syndrome are craniofacial disproportion with a microcephalic aspect, accompanied by calcifications (predominantly at the cortical-subcortical junction), malformations of cortical development, ventriculomegaly, and abnormalities in the formation of the corpus callosum. However, attention should be paid to the spectrum of potential presentations of congenital Zika syndrome, and the possibility of ZIKV involvement should not be ruled out when microcephaly is not present or when the neuroimaging findings are more subtle.
